# Di-μ-oxido-bis­({2-[(*R*,*R*)-(−)-(2-amino­cyclo­hexyl)imino­meth­yl]-4-nitro­phenolato-κ^3^ *N*,*N*′,*O*}oxidovanadium(V)) dimethyl sulfoxide disolvate

**DOI:** 10.1107/S1600536808038762

**Published:** 2008-12-10

**Authors:** Grzegorz Romanowski, Michał Wera, Artur Sikorski

**Affiliations:** a University of Gdańsk, Faculty of Chemistry, Sobieskiego 18/19, 80-952 Gdańsk, Poland

## Abstract

The title compound, [V_2_(C_13_H_16_N_3_O_3_)_2_O_4_]·2C_2_H_6_OS, is a centrosymmetric dimeric complex solvated by two dimethyl sulfoxide mol­ecules. Each V^V^ atom is six-coordinated by one oxide group, two N atoms and one O atom from the tridentate Schiff base ligand, and by two additional bridging O atoms in a distorted octa­hedral coordination geometry. Three atoms of the cyclo­hexane ring are each disordered over two sites, with occupancy factors of 0.501 (10) and 0.499 (10). C—H⋯O and N—H⋯O hydrogen bonds link the dimers and solvent mol­ecules into a supra­molecular network.

## Related literature

For general background, see: Carter-Franklin *et al.* (2003 ▶); Eady (2003 ▶); Evangelou (2002 ▶); Mendz (1991 ▶); Parekh *et al.* (2006 ▶); Rao *et al.* (1981 ▶); Rehder *et al.* (2002 ▶, 2003 ▶); Shahzadi *et al.* (2007 ▶). For related structures, see: Kwiatkowski *et al.* (2007 ▶); Mokry & Carrano (1993 ▶); Romanowski *et al.* (2008 ▶); Root *et al.* (1993 ▶). For the synthesis, see: Kwiatkowski *et al.* (2003 ▶).
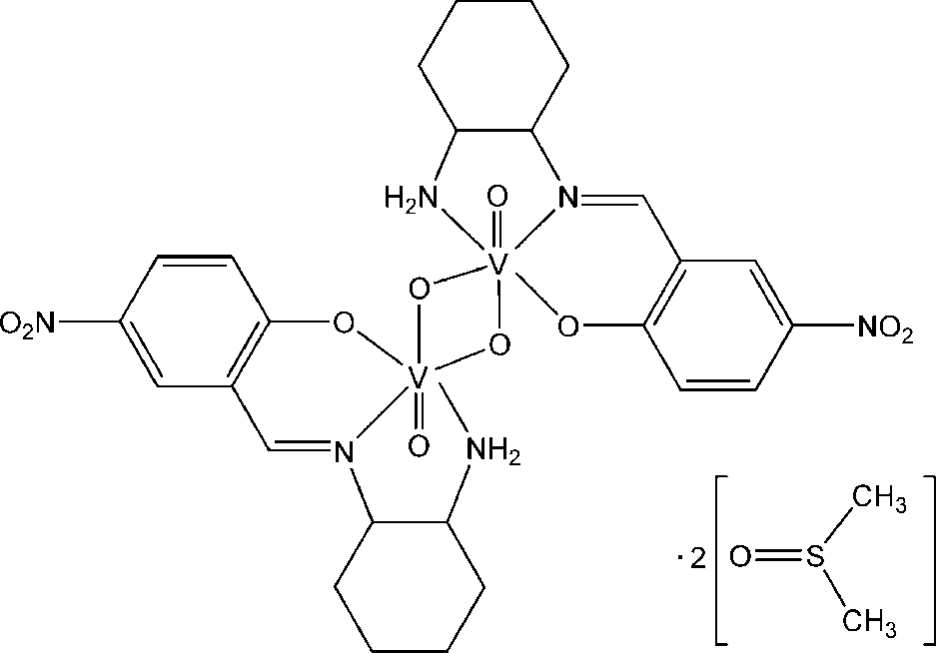


## Experimental

### 

#### Crystal data

[V_2_(C_13_H_16_N_3_O_3_)_2_O_4_]·2C_2_H_6_OS
*M* *_r_* = 846.71
Triclinic, 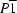

*a* = 7.249 (1) Å
*b* = 11.747 (2) Å
*c* = 11.809 (2) Å
α = 77.69 (3)°
β = 88.62 (3)°
γ = 76.13 (3)°
*V* = 953.4 (3) Å^3^
*Z* = 1
Mo *K*α radiation
μ = 0.67 mm^−1^
*T* = 295 (2) K
0.60 × 0.25 × 0.10 mm

#### Data collection

Oxford Diffraction Ruby CCD diffractometer
Absorption correction: multi-scan (*CrysAlis RED*; Oxford Diffraction, 2006 ▶) *T*_min_ = 0.720, *T*_max_ = 0.936
6786 measured reflections
3355 independent reflections
3245 reflections with *I* > 2σ(*I*)
*R*_int_ = 0.021

#### Refinement

*R*[*F* ^2^ > 2σ(*F* ^2^)] = 0.079
*wR*(*F* ^2^) = 0.194
*S* = 1.44
3355 reflections
256 parameters
H-atom parameters constrained
Δρ_max_ = 0.53 e Å^−3^
Δρ_min_ = −0.44 e Å^−3^

### 

Data collection: *CrysAlis CCD* (Oxford Diffraction, 2006 ▶); cell refinement: *CrysAlis RED* (Oxford Diffraction, 2006 ▶); data reduction: *CrysAlis RED*; program(s) used to solve structure: *SHELXS97* (Sheldrick, 2008 ▶); program(s) used to refine structure: *SHELXL97* (Sheldrick, 2008 ▶); molecular graphics: *ORTEPII* (Johnson, 1976 ▶); software used to prepare material for publication: *SHELXL97* and *PLATON* (Spek, 2003 ▶).

## Supplementary Material

Crystal structure: contains datablocks I, global. DOI: 10.1107/S1600536808038762/hy2164sup1.cif

Structure factors: contains datablocks I. DOI: 10.1107/S1600536808038762/hy2164Isup2.hkl

Additional supplementary materials:  crystallographic information; 3D view; checkCIF report

## Figures and Tables

**Table 1:** Selected bond lengths (Å)

N11—V19	2.186 (5)
N18—V19	2.109 (5)
V19—O20	1.610 (4)
V19—O21^i^	1.663 (4)
V19—O22	1.929 (4)
V19—O21	2.372 (4)

Symmetry code: (i) 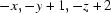.

**Table 2:** Hydrogen-bond geometry (Å, °)

*D*—H⋯*A*	*D*—H	H⋯*A*	*D*⋯*A*	*D*—H⋯*A*
N18—H18*A*⋯O22^i^	0.90	2.29	3.069 (6)	145
N18—H18*B*⋯O26^ii^	0.90	2.16	2.913 (8)	140
C5—H5*A*⋯O26^iii^	0.93	2.57	3.494 (9)	171
C10—H10*A*⋯O21^iv^	0.93	2.49	3.167 (7)	130
C13—H13*B*⋯O21^iv^	0.97	2.55	3.392 (7)	145
C14—H14*A*⋯O26^v^	0.97	2.38	3.211 (11)	144
C15—H15*B*⋯O8^vi^	0.97	2.32	3.109 (17)	138

Symmetry codes: (i) 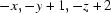; (ii) 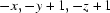; (iii) 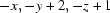; (iv) 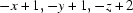; (v) 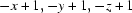; (vi) 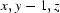.

**Fig. 1.**
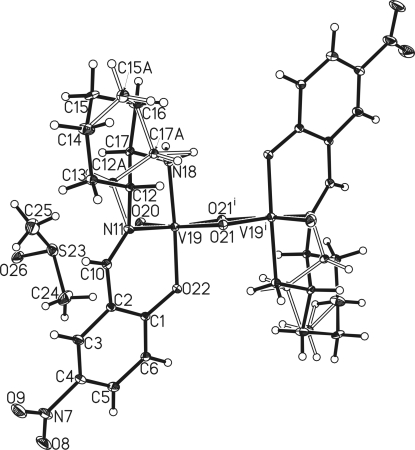
The molecular structure of the title compound, with displacement ellipsoids drawn at the 25% probability level. Disordered parts are shown by open bonds. [Symmetry code: (i) -x, -y + 1, -z + 2.]

**Fig. 2.**
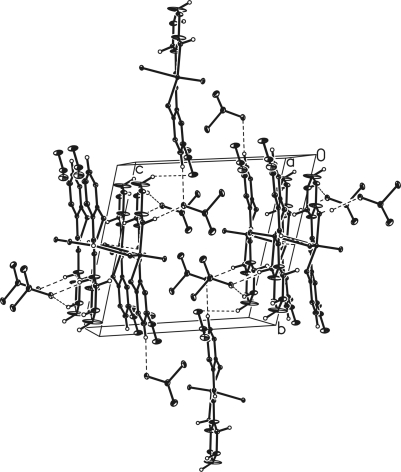
The arrangement of molecules in the title compound, viewed approximately along the a axis. Hydrogen bonds are represented by dashed lines.

## supplementary crystallographic information

### Comment

Research in vanadium chemistry and biochemistry increased after discovery of insulin mimetic (Rehder *et al.*, 2002), anticancer (Evangelou, 2002), antifungal and antibacterial (Parekh *et al.*, 2006; Shahzadi *et al.*, 2007) activity of this element. Oxidovanadium(IV) and (V) compounds exert catalytic activity like some biological enzymes, *viz*. haloperoxidases (Carter-Franklin *et al.*, 2003; Rehder *et al.*, 2003), phosphomutases (Mendz, 1991) and nitrogenases (Eady, 2003). Moreover, investigation of vanadium(V) complexes with Schiff bases is prompted by the fact that these ligands are coordinated to the metal through O and N atoms, similar to the coordination environments of natural systems.

The title compound was earlier characterized by spectroscopic methods (IR, UV-Vis, ^1^H and ^51^V NMR) (Kwiatkowski *et al.*, 2007). The crystal structure consists of a centrosymmetric dimeric vanadium(V) complex and two dimethyl sulfoxide (DMSO) molecules (Fig. 1). The singly deprotonated Schiff base acts as a tridentate ligand, forming one five- and one six-membered chelate rings. Each V^V^ atom is six-coordinated in a distorted octahedral environment. Two axial positions are occupied by one phenolate O atom (O22) and one amine N atom (N18), and the equatorial positions are occupied by one azomethine N atom (N11), two strongly [O20^i^ and O21^i^; symmetry code: (i) -*x*, -*y* + 1, -*z* + 2] and one weakly (O21) bonded oxide groups (Fig. 1). The V19—O20, V19—O21^i^(bridging) and V19—O22(phenolate) bond distances (Table 1) agree well with the corresponding values reported for related compounds (Mokry & Carrano, 1993; Romanowski *et al.*, 2008; Root *et al.*, 1993). The V19—O21 bond is longer than O21—V19^i^ bond due to the involvement of this O atom in bridging between the V atoms. The V19, O21, V19^i^, O21^i^ atoms are situated in the vertices of a parallelogram with the acute O21—V19—O21^i^ angle of 77.9 (2)°. The V···V separation is 3.170 (1)Å and falls within the range of known V···V distances in double-bridged vanadium polynuclear systems (Mokry & Carrano, 1993; Romanowski *et al.*, 2008; Root *et al.*, 1993). Three C atoms of the cyclohexane ring exhibit twofold disorder. The C12, C15 and C17 atoms are each disordered over two sites, with occupancy factors of 0.501 (10) and 0.499 (10). The five-membered chelate ring defined by V19, N11, C12, C17, N18 adopts a twisted conformation on C12 and C17 atoms, with *P* = 250.3 (5)° and τ_(_*_M_*_)_ = 56.7 (6)° for reference bond V19—N11 (Rao *et al.*, 1981) (Fig. 1).

In the crystal structure, C—H···O and N—H···O hydrogen bonds link the dimers and solvent molecules into a supramolecular network (Table 2; Fig. 2).

### Experimental

The title compound was obtained in a template/complexation reaction analogous to that described for preparation of dioxidovanadium(V) complexes with Schiff base ligands (Kwiatkowski *et al.*, 2003). A solution of *R,R*-(–)-1,2-diaminocyclohexane (1 mmol) in absolute ethanol (10 ml) was added under stirring to a freshly filtered solution of vanadium(V) oxytriethoxide (1 mmol) in absolute ethanol (50 ml), producing a yellow suspension of the intermediate. 5-Nitrosalicylaldehyde (1 mmol) dissolved in absolute ethanol was added to the aforementioned suspension. After refluxing of the resulting mixture (70 ml) for 2 h and its cooling to room temperature, the separated solids were filtered off, washed several times with ethanol, recrystallized from DMSO–EtOH mixture and dried over molecular sieves.

### Refinement

All H atoms were positioned geometrically and refined using a riding model, with C—H = 0.93–0.98 and N—H = 0.90 Å, and with *U*_iso_(H) = 1.2(1.5 for methyl group)*U*_eq_(C,*N*).

### Crystal data

**Table:**

[V_2_(C_13_H_16_N_3_O_3_)_2_O_4_]·2C_2_H_6_OS	*Z* = 1
*M**_r_* = 846.71	*F*(000) = 440
Triclinic, *P*1	*D*_x_ = 1.475 Mg m^−^^3^
Hall symbol: -P 1	Mo *K*α radiation, λ = 0.71073 Å
*a* = 7.249 (1) Å	Cell parameters from 3355 reflections
*b* = 11.747 (2) Å	θ = 3.0–25.1°
*c* = 11.809 (2) Å	µ = 0.67 mm^−^^1^
α = 77.69 (3)°	*T* = 295 K
β = 88.62 (3)°	Needle, yellow
γ = 76.13 (3)°	0.60 × 0.25 × 0.10 mm
*V* = 953.4 (3) Å^3^	

### Data collection

**Table:**

Oxford Diffraction Ruby CCD diffractometer	3355 independent reflections
Radiation source: Enhance (Mo) X-ray Source	3245 reflections with *I* > 2σ(*I*)
graphite	*R*_int_ = 0.021
Detector resolution: 10.4002 pixels mm^-1^	θ_max_ = 25.1°, θ_min_ = 3.0°
ω scans	*h* = −8→8
Absorption correction: multi-scan (*CrysAlis RED*; Oxford Diffraction, 2006)	*k* = −13→11
*T*_min_ = 0.720, *T*_max_ = 0.936	*l* = −13→14
6786 measured reflections	

### Refinement

**Table:**

Refinement on *F*^2^	Secondary atom site location: difference Fourier map
Least-squares matrix: full	Hydrogen site location: inferred from neighbouring sites
*R*[*F*^2^ > 2σ(*F*^2^)] = 0.079	H-atom parameters constrained
*wR*(*F*^2^) = 0.194	*w* = 1/[σ^2^(*F*_o_^2^) + (0.0232*P*)^2^ + 4.2049*P*] where *P* = (*F*_o_^2^ + 2*F*_c_^2^)/3
*S* = 1.44	(Δ/σ)_max_ < 0.001
3355 reflections	Δρ_max_ = 0.53 e Å^−^^3^
256 parameters	Δρ_min_ = −0.44 e Å^−^^3^
0 restraints	Extinction correction: *SHELXL97* (Sheldrick, 2008), Fc^*^=kFc[1+0.001xFc^2^λ^3^/sin(2θ)]^-1/4^
Primary atom site location: structure-invariant direct methods	Extinction coefficient: 0.0061 (16)

### Fractional atomic coordinates and isotropic or equivalent isotropic displacement parameters (Å^2^)

**Table:**

	*x*	*y*	*z*	*U*_iso_*/*U*_eq_	Occ. (<1)
C1	0.1768 (8)	0.7547 (5)	0.8238 (5)	0.0318 (12)	
C2	0.3625 (8)	0.7030 (5)	0.7934 (5)	0.0322 (12)	
C3	0.4761 (10)	0.7777 (6)	0.7378 (6)	0.0448 (15)	
H3A	0.5999	0.7450	0.7188	0.054*	
C4	0.4032 (11)	0.8999 (6)	0.7112 (6)	0.0505 (17)	
C5	0.2207 (11)	0.9522 (6)	0.7375 (6)	0.0525 (18)	
H5A	0.1727	1.0348	0.7155	0.063*	
C6	0.1108 (10)	0.8802 (5)	0.7969 (5)	0.0403 (14)	
H6A	−0.0095	0.9151	0.8197	0.048*	
N7	0.5242 (12)	0.9771 (6)	0.6526 (7)	0.077 (2)	
O8	0.4474 (11)	1.0817 (5)	0.6099 (7)	0.103 (3)	
O9	0.6933 (10)	0.9331 (6)	0.6482 (8)	0.101 (3)	
C10	0.4411 (8)	0.5748 (5)	0.8148 (5)	0.0335 (13)	
H10A	0.5713	0.5473	0.8071	0.040*	
N11	0.3421 (7)	0.4965 (4)	0.8437 (4)	0.0329 (11)	
C12	0.436 (2)	0.3677 (13)	0.8877 (13)	0.030 (3)	0.501 (10)
H12A	0.4263	0.3496	0.9722	0.036*	0.501 (10)
C12A	0.428 (2)	0.3682 (13)	0.8308 (15)	0.032 (3)	0.499 (10)
H12B	0.4040	0.3614	0.7515	0.038*	0.499 (10)
C13	0.6421 (8)	0.3255 (5)	0.8594 (6)	0.0405 (14)	
H13A	0.6607	0.3629	0.7798	0.049*	
H13B	0.7202	0.3522	0.9090	0.049*	
C14	0.7089 (11)	0.1916 (6)	0.8742 (9)	0.070 (2)	
H14A	0.8251	0.1749	0.8318	0.083*	
H14B	0.7415	0.1579	0.9557	0.083*	
C15	0.5840 (19)	0.1297 (11)	0.8390 (14)	0.048 (4)	0.501 (10)
H15A	0.6353	0.0441	0.8656	0.058*	0.501 (10)
H15B	0.5754	0.1470	0.7550	0.058*	0.501 (10)
C15A	0.595 (2)	0.1146 (13)	0.9356 (14)	0.054 (4)*	0.499 (10)
H15C	0.6012	0.1126	1.0180	0.064*	0.499 (10)
H15D	0.6433	0.0335	0.9240	0.064*	0.499 (10)
C16	0.3804 (10)	0.1654 (6)	0.8875 (8)	0.057 (2)	
H16A	0.3852	0.1485	0.9715	0.069*	
H16B	0.2972	0.1216	0.8621	0.069*	
C17	0.3085 (16)	0.3038 (10)	0.8368 (11)	0.032 (3)	0.501 (10)
H17A	0.3097	0.3219	0.7519	0.038*	0.501 (10)
C17A	0.3189 (16)	0.2942 (10)	0.9138 (13)	0.033 (3)*	0.499 (10)
H17B	0.3453	0.2941	0.9948	0.040*	0.499 (10)
N18	0.1126 (7)	0.3474 (4)	0.8793 (4)	0.0361 (11)	
H18A	0.1072	0.3108	0.9541	0.043*	
H18B	0.0280	0.3264	0.8382	0.043*	
V19	0.03456 (13)	0.53484 (9)	0.86534 (8)	0.0289 (3)	
O20	−0.0303 (7)	0.5634 (4)	0.7308 (4)	0.0490 (12)	
O21	0.1601 (5)	0.4698 (4)	1.0582 (3)	0.0325 (9)	
O22	0.0653 (6)	0.6894 (3)	0.8812 (3)	0.0364 (9)	
S23	−0.0005 (3)	0.6916 (2)	0.44455 (18)	0.0657 (6)	
C24	0.0490 (17)	0.8021 (9)	0.5086 (8)	0.094 (3)	
H24A	−0.0272	0.8791	0.4711	0.140*	
H24B	0.1811	0.8019	0.5006	0.140*	
H24C	0.0202	0.7860	0.5894	0.140*	
C25	0.2180 (17)	0.5849 (10)	0.4774 (10)	0.104 (4)	
H25A	0.2351	0.5309	0.4253	0.156*	
H25B	0.2182	0.5404	0.5557	0.156*	
H25C	0.3197	0.6252	0.4693	0.156*	
O26	−0.0092 (10)	0.7404 (5)	0.3172 (5)	0.0747 (17)	

### Atomic displacement parameters (Å^2^)

**Table:**

	*U*^11^	*U*^22^	*U*^33^	*U*^12^	*U*^13^	*U*^23^
C1	0.038 (3)	0.034 (3)	0.028 (3)	−0.015 (2)	−0.004 (2)	−0.008 (2)
C2	0.040 (3)	0.033 (3)	0.027 (3)	−0.016 (2)	−0.003 (2)	−0.006 (2)
C3	0.045 (4)	0.040 (4)	0.053 (4)	−0.019 (3)	0.003 (3)	−0.009 (3)
C4	0.065 (5)	0.036 (3)	0.055 (4)	−0.024 (3)	0.014 (3)	−0.006 (3)
C5	0.066 (5)	0.033 (3)	0.057 (4)	−0.013 (3)	0.004 (4)	−0.006 (3)
C6	0.051 (4)	0.033 (3)	0.038 (3)	−0.011 (3)	0.002 (3)	−0.008 (3)
N7	0.081 (5)	0.046 (4)	0.109 (6)	−0.033 (4)	0.030 (5)	−0.011 (4)
O8	0.115 (6)	0.048 (4)	0.140 (7)	−0.034 (4)	0.046 (5)	0.007 (4)
O9	0.081 (5)	0.072 (4)	0.156 (7)	−0.041 (4)	0.047 (5)	−0.016 (4)
C10	0.029 (3)	0.038 (3)	0.034 (3)	−0.012 (2)	−0.002 (2)	−0.007 (2)
N11	0.031 (2)	0.028 (2)	0.040 (3)	−0.011 (2)	−0.003 (2)	−0.003 (2)
C12	0.035 (7)	0.030 (7)	0.026 (8)	−0.010 (5)	−0.002 (7)	−0.008 (7)
C12A	0.031 (7)	0.033 (7)	0.034 (9)	−0.008 (5)	−0.003 (7)	−0.011 (7)
C13	0.034 (3)	0.038 (3)	0.050 (4)	−0.011 (3)	−0.003 (3)	−0.008 (3)
C14	0.040 (4)	0.040 (4)	0.121 (8)	−0.002 (3)	0.001 (4)	−0.008 (4)
C15	0.053 (8)	0.028 (6)	0.064 (10)	0.003 (6)	−0.013 (7)	−0.023 (6)
C16	0.050 (4)	0.036 (4)	0.093 (6)	−0.017 (3)	0.004 (4)	−0.021 (4)
C17	0.039 (6)	0.032 (6)	0.027 (7)	−0.010 (5)	0.001 (5)	−0.013 (5)
N18	0.032 (3)	0.042 (3)	0.039 (3)	−0.015 (2)	0.001 (2)	−0.009 (2)
V19	0.0284 (5)	0.0349 (6)	0.0256 (5)	−0.0116 (4)	−0.0012 (4)	−0.0062 (4)
O20	0.061 (3)	0.053 (3)	0.028 (2)	−0.007 (2)	−0.009 (2)	−0.005 (2)
O21	0.029 (2)	0.041 (2)	0.031 (2)	−0.0135 (17)	−0.0036 (16)	−0.0099 (17)
O22	0.042 (2)	0.033 (2)	0.037 (2)	−0.0149 (18)	0.0058 (18)	−0.0062 (17)
S23	0.0761 (14)	0.0722 (14)	0.0477 (11)	−0.0224 (11)	0.0021 (10)	−0.0052 (10)
C24	0.125 (9)	0.093 (7)	0.062 (6)	−0.005 (6)	−0.013 (6)	−0.038 (5)
C25	0.121 (10)	0.086 (7)	0.080 (7)	0.006 (7)	0.012 (7)	0.003 (6)
O26	0.114 (5)	0.066 (4)	0.043 (3)	−0.014 (3)	0.001 (3)	−0.020 (3)

### Geometric parameters (Å, °)

**Table:**

C1—O22	1.323 (7)	C15—C16	1.564 (16)
C1—C6	1.405 (8)	C15—H15A	0.9700
C1—C2	1.410 (8)	C15—H15B	0.9700
C2—C3	1.397 (8)	C15A—C16	1.599 (16)
C2—C10	1.446 (8)	C15A—H15C	0.9700
C3—C4	1.375 (9)	C15A—H15D	0.9700
C3—H3A	0.9300	C16—C17A	1.567 (13)
C4—C5	1.377 (10)	C16—C17	1.570 (13)
C4—N7	1.468 (9)	C16—H16A	0.9700
C5—C6	1.373 (9)	C16—H16B	0.9700
C5—H5A	0.9300	C17—N18	1.503 (12)
C6—H6A	0.9300	C17—H17A	0.9800
N7—O9	1.215 (10)	C17A—N18	1.505 (12)
N7—O8	1.222 (9)	C17A—H17B	0.9800
C10—N11	1.284 (7)	N18—V19	2.109 (5)
C10—H10A	0.9300	N18—H18A	0.9000
N11—C12	1.487 (15)	N18—H18B	0.9000
N11—C12A	1.526 (15)	V19—O20	1.610 (4)
N11—V19	2.186 (5)	V19—O21^i^	1.663 (4)
C12—C13	1.507 (16)	V19—O22	1.929 (4)
C12—C17	1.529 (18)	V19—O21	2.372 (4)
C12—H12A	0.9800	O21—V19^i^	1.663 (4)
C12A—C17A	1.51 (2)	S23—O26	1.489 (6)
C12A—C13	1.534 (16)	S23—C24	1.744 (10)
C12A—H12B	0.9800	S23—C25	1.761 (11)
C13—C14	1.503 (9)	C24—H24A	0.9600
C13—H13A	0.9700	C24—H24B	0.9600
C13—H13B	0.9700	C24—H24C	0.9600
C14—C15	1.409 (15)	C25—H25A	0.9600
C14—C15A	1.439 (16)	C25—H25B	0.9600
C14—H14A	0.9700	C25—H25C	0.9600
C14—H14B	0.9700		
			
O22—C1—C6	118.8 (5)	C14—C15A—H15D	110.0
O22—C1—C2	122.3 (5)	C16—C15A—H15D	110.0
C6—C1—C2	118.9 (5)	H15C—C15A—H15D	108.3
C3—C2—C1	119.3 (5)	C15—C16—C17A	116.3 (7)
C3—C2—C10	117.8 (6)	C15—C16—C17	105.1 (8)
C1—C2—C10	122.9 (5)	C17A—C16—C15A	104.9 (9)
C4—C3—C2	119.5 (6)	C17—C16—C15A	118.5 (8)
C4—C3—H3A	120.3	C15—C16—H16A	110.7
C2—C3—H3A	120.3	C17A—C16—H16A	77.7
C3—C4—C5	122.2 (6)	C17—C16—H16A	110.7
C3—C4—N7	118.8 (7)	C15A—C16—H16A	69.4
C5—C4—N7	119.0 (6)	C15—C16—H16B	110.7
C6—C5—C4	118.8 (6)	C17A—C16—H16B	126.2
C6—C5—H5A	120.6	C17—C16—H16B	110.7
C4—C5—H5A	120.6	C15A—C16—H16B	127.8
C5—C6—C1	121.1 (6)	H16A—C16—H16B	108.8
C5—C6—H6A	119.4	N18—C17—C12	106.1 (9)
C1—C6—H6A	119.4	N18—C17—C16	109.3 (8)
O9—N7—O8	124.1 (7)	C12—C17—C16	107.9 (9)
O9—N7—C4	118.3 (7)	N18—C17—H17A	111.1
O8—N7—C4	117.6 (8)	C12—C17—H17A	111.1
N11—C10—C2	124.0 (5)	C16—C17—H17A	111.1
N11—C10—H10A	118.0	N18—C17A—C12A	105.7 (10)
C2—C10—H10A	118.0	N18—C17A—C16	109.3 (8)
C10—N11—C12	120.6 (7)	C12A—C17A—C16	104.9 (10)
C10—N11—C12A	118.6 (7)	N18—C17A—H17B	112.2
C10—N11—V19	125.9 (4)	C12A—C17A—H17B	112.2
C12—N11—V19	112.6 (6)	C16—C17A—H17B	112.2
C12A—N11—V19	113.3 (6)	C17—N18—V19	113.2 (5)
N11—C12—C13	117.7 (10)	C17A—N18—V19	112.5 (5)
N11—C12—C17	102.5 (10)	C17—N18—H18A	108.9
C13—C12—C17	112.1 (11)	C17A—N18—H18A	77.3
N11—C12—H12A	108.1	V19—N18—H18A	108.9
C13—C12—H12A	108.1	C17—N18—H18B	108.9
C17—C12—H12A	108.1	C17A—N18—H18B	133.8
C17A—C12A—N11	103.9 (11)	V19—N18—H18B	108.9
C17A—C12A—C13	110.9 (11)	H18A—N18—H18B	107.8
N11—C12A—C13	113.7 (10)	O20—V19—O21^i^	106.7 (2)
C17A—C12A—H12B	109.4	O20—V19—O22	101.0 (2)
N11—C12A—H12B	109.4	O21^i^—V19—O22	99.26 (19)
C13—C12A—H12B	109.4	O20—V19—N18	94.0 (2)
C14—C13—C12	113.9 (7)	O21^i^—V19—N18	93.45 (19)
C14—C13—C12A	111.2 (7)	O22—V19—N18	156.55 (19)
C14—C13—H13A	108.8	O20—V19—N11	98.4 (2)
C12—C13—H13A	108.8	O21^i^—V19—N11	153.60 (19)
C12A—C13—H13A	86.8	O22—V19—N11	83.61 (18)
C14—C13—H13B	108.8	N18—V19—N11	76.42 (18)
C12—C13—H13B	108.8	O20—V19—O21	172.2 (2)
C12A—C13—H13B	129.9	O21^i^—V19—O21	77.86 (17)
H13A—C13—H13B	107.7	O22—V19—O21	84.18 (16)
C15—C14—C15A	46.2 (9)	N18—V19—O21	79.31 (17)
C15—C14—C13	118.0 (8)	N11—V19—O21	76.33 (16)
C15A—C14—C13	120.1 (9)	V19^i^—O21—V19	102.14 (17)
C15—C14—H14A	107.8	C1—O22—V19	129.1 (4)
C15A—C14—H14A	131.9	O26—S23—C24	106.2 (4)
C13—C14—H14A	107.8	O26—S23—C25	107.2 (5)
C15—C14—H14B	107.8	C24—S23—C25	98.3 (6)
C15A—C14—H14B	63.4	S23—C24—H24A	109.5
C13—C14—H14B	107.8	S23—C24—H24B	109.5
H14A—C14—H14B	107.2	H24A—C24—H24B	109.5
C14—C15—C16	112.3 (9)	S23—C24—H24C	109.5
C14—C15—H15A	109.1	H24A—C24—H24C	109.5
C16—C15—H15A	109.1	H24B—C24—H24C	109.5
C14—C15—H15B	109.1	S23—C25—H25A	109.5
C16—C15—H15B	109.1	S23—C25—H25B	109.5
H15A—C15—H15B	107.9	H25A—C25—H25B	109.5
C14—C15A—C16	108.7 (10)	S23—C25—H25C	109.5
C14—C15A—H15C	110.0	H25A—C25—H25C	109.5
C16—C15A—H15C	110.0	H25B—C25—H25C	109.5
			
O22—C1—C2—C3	−176.9 (5)	C15—C16—C17—N18	−179.6 (8)
C6—C1—C2—C3	0.2 (8)	C17A—C16—C17—N18	−64.4 (11)
O22—C1—C2—C10	4.5 (8)	C15A—C16—C17—N18	−137.2 (10)
C6—C1—C2—C10	−178.4 (5)	C15—C16—C17—C12	−64.7 (12)
C1—C2—C3—C4	−1.6 (9)	C17A—C16—C17—C12	50.5 (11)
C10—C2—C3—C4	177.1 (6)	C15A—C16—C17—C12	−22.3 (14)
C2—C3—C4—C5	−0.1 (11)	N11—C12A—C17A—N18	55.5 (12)
C2—C3—C4—N7	−179.9 (7)	C13—C12A—C17A—N18	178.1 (9)
C3—C4—C5—C6	3.0 (12)	N11—C12A—C17A—C16	170.9 (9)
N7—C4—C5—C6	−177.2 (7)	C13—C12A—C17A—C16	−66.5 (13)
C4—C5—C6—C1	−4.4 (11)	C15—C16—C17A—N18	141.3 (9)
O22—C1—C6—C5	−180.0 (6)	C17—C16—C17A—N18	64.3 (12)
C2—C1—C6—C5	2.8 (9)	C15A—C16—C17A—N18	−176.0 (9)
C3—C4—N7—O9	−13.0 (13)	C15—C16—C17A—C12A	28.3 (14)
C5—C4—N7—O9	167.2 (9)	C17—C16—C17A—C12A	−48.7 (12)
C3—C4—N7—O8	166.1 (8)	C15A—C16—C17A—C12A	71.0 (12)
C5—C4—N7—O8	−13.7 (13)	C12—C17—N18—C17A	−51.4 (11)
C3—C2—C10—N11	−165.8 (6)	C16—C17—N18—C17A	64.7 (11)
C1—C2—C10—N11	12.8 (9)	C12—C17—N18—V19	45.1 (10)
C2—C10—N11—C12	−167.3 (8)	C16—C17—N18—V19	161.1 (6)
C2—C10—N11—C12A	163.0 (8)	C12A—C17A—N18—C17	47.5 (11)
C2—C10—N11—V19	1.0 (8)	C16—C17A—N18—C17	−64.9 (12)
C10—N11—C12—C13	−20.5 (15)	C12A—C17A—N18—V19	−51.1 (11)
C12A—N11—C12—C13	73 (2)	C16—C17A—N18—V19	−163.6 (6)
V19—N11—C12—C13	169.7 (8)	C17—N18—V19—O20	82.6 (6)
C10—N11—C12—C17	−144.0 (8)	C17A—N18—V19—O20	120.2 (7)
C12A—N11—C12—C17	−50.9 (19)	C17—N18—V19—O21^i^	−170.4 (6)
V19—N11—C12—C17	46.2 (11)	C17A—N18—V19—O21^i^	−132.7 (7)
C10—N11—C12A—C17A	157.6 (8)	C17—N18—V19—O22	−47.4 (8)
C12—N11—C12A—C17A	56 (2)	C17A—N18—V19—O22	−9.8 (9)
V19—N11—C12A—C17A	−38.3 (12)	C17—N18—V19—N11	−15.1 (6)
C10—N11—C12A—C13	36.8 (14)	C17A—N18—V19—N11	22.6 (6)
C12—N11—C12A—C13	−65 (2)	C17—N18—V19—O21	−93.4 (6)
V19—N11—C12A—C13	−159.0 (8)	C17A—N18—V19—O21	−55.8 (7)
N11—C12—C13—C14	−162.4 (9)	C10—N11—V19—O20	80.1 (5)
C17—C12—C13—C14	−43.9 (13)	C12—N11—V19—O20	−110.8 (7)
N11—C12—C13—C12A	−73 (2)	C12A—N11—V19—O20	−82.7 (8)
C17—C12—C13—C12A	45.4 (18)	C10—N11—V19—O21^i^	−118.1 (6)
C17A—C12A—C13—C14	49.1 (14)	C12—N11—V19—O21^i^	51.0 (9)
N11—C12A—C13—C14	165.8 (9)	C12A—N11—V19—O21^i^	79.1 (9)
C17A—C12A—C13—C12	−52 (2)	C10—N11—V19—O22	−20.1 (5)
N11—C12A—C13—C12	64.4 (19)	C12—N11—V19—O22	149.0 (7)
C12—C13—C14—C15	39.7 (14)	C12A—N11—V19—O22	177.1 (7)
C12A—C13—C14—C15	12.1 (14)	C10—N11—V19—N18	172.2 (5)
C12—C13—C14—C15A	−13.6 (14)	C12—N11—V19—N18	−18.6 (7)
C12A—C13—C14—C15A	−41.2 (14)	C12A—N11—V19—N18	9.5 (7)
C15A—C14—C15—C16	57.2 (12)	C10—N11—V19—O21	−105.7 (5)
C13—C14—C15—C16	−48.9 (15)	C12—N11—V19—O21	63.5 (7)
C15—C14—C15A—C16	−53.4 (10)	C12A—N11—V19—O21	91.6 (7)
C13—C14—C15A—C16	47.8 (15)	O21^i^—V19—O21—V19^i^	0.000 (2)
C14—C15—C16—C17A	27.0 (15)	O22—V19—O21—V19^i^	100.8 (2)
C14—C15—C16—C17	60.4 (13)	N18—V19—O21—V19^i^	−95.9 (2)
C14—C15—C16—C15A	−55.7 (12)	N11—V19—O21—V19^i^	−174.4 (2)
C14—C15A—C16—C15	52.1 (11)	C6—C1—O22—V19	144.0 (4)
C14—C15A—C16—C17A	−60.9 (13)	C2—C1—O22—V19	−38.9 (7)
C14—C15A—C16—C17	−28.2 (15)	O20—V19—O22—C1	−58.2 (5)
N11—C12—C17—N18	−57.2 (11)	O21^i^—V19—O22—C1	−167.4 (5)
C13—C12—C17—N18	175.7 (9)	N18—V19—O22—C1	70.7 (7)
N11—C12—C17—C16	−174.2 (8)	N11—V19—O22—C1	39.1 (5)
C13—C12—C17—C16	58.7 (13)	O21—V19—O22—C1	116.0 (5)

Symmetry codes: (i) −*x*, −*y*+1, −*z*+2.

### Hydrogen-bond geometry (Å, °)

**Table:**

*D*—H···*A*	*D*—H	H···*A*	*D*···*A*	*D*—H···*A*
N18—H18A···O22^i^	0.90	2.29	3.069 (6)	145
N18—H18B···O26^ii^	0.90	2.16	2.913 (8)	140
C5—H5A···O26^iii^	0.93	2.57	3.494 (9)	171
C10—H10A···O21^iv^	0.93	2.49	3.167 (7)	130
C13—H13B···O21^iv^	0.97	2.55	3.392 (7)	145
C14—H14A···O26^v^	0.97	2.38	3.211 (11)	144
C15—H15B···O8^vi^	0.97	2.32	3.109 (17)	138

Symmetry codes:  (i) −*x*, −*y*+1, −*z*+2; (ii) −*x*, −*y*+1, −*z*+1; (iii) −*x*, −*y*+2, −*z*+1; (iv) −*x*+1, −*y*+1, −*z*+2; (v) −*x*+1, −*y*+1, −*z*+1; (vi) *x*, *y*−1, *z*.
